# Oneida County Veterinary Medical Society

**Published:** 1897-04

**Authors:** J. M. Currie

**Affiliations:** Secretary


### ONEIDA COUNTY VETERINARY MEDICAL SOCIETY.

In the parlors of Stanwix Hall, Rome, N. Y., February 2, 1897, was held the regular quarterly meeting. Called to order by Dr. F. Morrow, President.

The following members responded to roll-call: Drs. F. Morrow, Oneida; A. Findlay, Camden; W. G. Hollingsworth, Utica; L. G. Moore, Trenton; R. C. Hurlburt, Boonville; H. W. Skerritt, Utica; H. D. Stebbins, West Winfield; Wilson Huff and J. M. Currie, Rome. The minutes of last meeting were read and approved.

When the order of new business was called, the incorporation of the Society was fully discussed. On motion, Dr. Hollingsworth was instructed to take legal steps to do so.

Dr. H. W. Pratt, Rome, was admitted to membership by a unanimous vote.

A communication from the State Board of Health was read, asking all the members of this Society to report all cases of tuberculosis in cattle that came under their notice; all agreed to do so.

Moved and seconded that the next meeting be held Tuesday, May 4, 1897, at the residence of Dr. Hollingsworth, 24 Summit Place, Utica, N. Y.; carried.

Next came the reading of papers: Dr. Findlay presented a paper on "Luxation of the Hip-joint in Cattle," relating much of his personal experience. A general and hearty discussion followed. Dr. Huff read a paper on "Advice to Veterinarians: The Opportunities of Educated Men." Also fully discussed.

There being no further business, an adjournment was in order. Essayist for next meeting, Dr. H. D. Stebbins.

J. M. Currie, V.S.,
Secretary.

